# Consuming High-Fat and Low-Fat Ground Beef Depresses High-Density and Low-Density Lipoprotein Cholesterol Concentrations, and Reduces Small, Dense Low-Density Lipoprotein Particle Abundance

**DOI:** 10.3390/nu15020337

**Published:** 2023-01-10

**Authors:** Jason R. Lytle, Tara Price, Stephen F. Crouse, Dana R. Smith, Rosemary L. Walzem, Stephen B. Smith

**Affiliations:** 1 Department of Health & Kinesiology, Texas A&M University, College Station, TX 77843, USA; 2 Department of Nutrition and Food Science, College Station, TX 77843, USA; 3 Independent Nutrition Consultant, College Station, TX 77845, USA; 4 Poultry Science, Texas A&M University, College Station, TX 77843, USA; 5 Graduate Faculty of Nutrition, Texas A&M University, College Station, TX 77843, USA; 6 Department of Animal Science, Texas A&M University, College Station, TX 77843, USA

**Keywords:** ground beef consumption, saturated fat intake, lipoprotein cholesterol

## Abstract

We hypothesized that consumption of high-fat (HF) ground beef (24% fat) would not affect plasma concentrations of high-density lipoprotein cholesterol (HDL-C) or low-density lipoprotein (LDL-C), whereas low-fat (LF) ground beef (5% fat) would decrease HDL-C and LDL-C concentrations. In a randomized 2-period crossover, controlled feeding trial, 25 men (mean age and body mass index, 40 years and 31.2) consumed 115-g HF or LF patties, 5/week for 5 weeks with a 4-week washout. The HF treatment increased % energy from fat (*p* = 0.006) and saturated fat (*p* = 0.004) and tended (*p* = 0.060) to depress % energy from carbohydrates. The HF and LF treatments decreased the plasma concentrations of HDL-C (*p* = 0.001) and LDL-C (*p* = 0.011). Both ground beef treatments decreased the abundance of HDL_3a_ and increased the abundance of HDL_3_ (*p* ≤ 0.003); the LF treatment also decreased the abundance of HDL_2b_ and HDL_2a_ (*p* ≤ 0.012). The HF and LF treatments decreased the abundance of LDL_3_ and LDL_4_ (*p* ≤ 0.024) and the HF treatment also decreased LDL_5_ (*p* = 0.041). Contrary to our hypothesis, the HF treatment decreased plasma HDL-C and LDL-C concentrations despite increased saturated fat intake, and both treatments decreased the abundance of smaller, denser LDL subfractions.

## 1. Introduction

Beef is a popular food worldwide, and the United States (U.S.) consumes 21% of the world’s beef production, with China, the European Union, and Brazil consuming 16%, 13%, and 13%, respectively, of the world’s production (https://www.fas.usda.gov/) (accessed on 2 December 2022). It is estimated that ground beef constitutes 40–45% of the beef consumed in the U.S.; when beef is prepared for meals at home, ground beef is used 60% of the time (https://beef2live.com/story-ground-beef-united-states-128-104332) (accessed on 2 December 2022). Ground beef containing less than 5–9% fat, 10% fat, and 20% fat accounts for 20, 42, and 20% of retail sales, respectively; the remaining 18% is comprised primarily of ground beef containing 30% fat (https://www.beefitswhatsfordinner.com/retail/sales-data-shopper-insights/ground-beef-at-retail-and-foodservice) (accessed on 2 December 2022). Palmitic acid (16:0) and stearic acid (18:0) are the most abundant saturated fatty acids (SFA) in beef, and oleic acid (18:1n-9) is the most abundant monounsaturated fatty acid (MUFA) in ground beef [1,2,3,4,5]. Beef also contains significant amounts of naturally occurring *trans*-fatty acid (TFA), primarily eladic acid (18:1*trans*-9) and *trans*-vaccenic acid (18:1*trans*-11), and conventional, 20% fat ground beef (22.8 g fat/114-g patty) contains 5 g palmitic acid, 3 g stearic acid, 8 g oleic acid, and 1.7 g TFA [1]. However, TFA derived from ruminal sources (e.g., dairy products and beef) does not affect the risk for cardiovascular disease (CVD) [6].

As indicated above, ground beef is a popular component in U.S. diet, and as such, ground beef constitutes one of the primary dietary sources of saturated fatty acids. In addition, the total fat and saturated fatty acid content can be changed readily and accurately during the formulation of ground beef preparations. Therefore, we conducted several randomized, controlled trials with men and/or postmenopausal women in which we tested the effects of ground beef differing in fatty acid composition on risk factors for CVD. Adams et al. [1] reported that high-density lipoprotein cholesterol (HDL-C) and low-density lipoprotein cholesterol (LDL-C) concentrations decreased from baseline when mildly hypercholesterolemic men were fed 35% fat, 114-g ground beef patties (40 g fat/patty; 15.7 g SFA/patty) (5 patties/week for 5 weeks). In a randomized controlled trial with normocholesterolemic men, consumption of 24% fat, 114-g patties (27 g fat/patty; 14.4 g SFA/patty) (5 patties/week for 5 weeks) had no effect on HDL-C or LDL-C concentration [2]. In a subsequent randomized controlled trial with postmenopausal women, consumption of 21% fat, 114-g patties (24 g fat/patty; 10.5 g SFA/patty) (5 patties/week for 6 weeks) had no effect on HDL-C or LDL-C concentration [3]. Choi et al. [4] reported that in a randomized controlled trial including postmenopausal women and older men, consumption of 22% fat, 114 g patties (25 g fat/patty; 10.5 g SFA/patty) (5 patties/week for 5 weeks) had no effect on HDL-C or LDL-C concentration. When data were pooled across the four trials (*n* = 42 men, 24 women), we established that intakes of 114-g high-fat/high-SFA ground beef patties 5 times per week for 5 or 6 weeks did not significantly affect HDL-C or LDL-C concentration [5]. 

Our previous studies also demonstrated that ground beef interventions elicit differences in lipoprotein particle sizes. LDL particle diameter decreased in men following a 35% fat ground beef intervention [1], and HDL_2_ and HDL_3_ particle diameter decreased in men following a 24% fat ground beef intervention [2]. In contrast to [1], LDL particle diameter increased in men and women following consumption of 25 g fat/patty ground beef, which was reflected in a greater concentration of cholesterol occurring in the LDL_1_ and LDL_2_ subfractions [4]. Because our previous studies indicated that ground beef consumption could affect lipoprotein particle size, a feature that varies inversely with flotation density, a secondary outcome of the current trial was to document the effects of LF (5% fat) and HF (25% fat) ground beef consumption on the density distributions for LDL and HDL subfractions. We previously determined particle density distributions using isopycnic density profiling of lipoproteins pre-stained with a lipophilic fluorescent probe [7]. In that method, the density distribution of labeled lipoproteins was analyzed as the area under the curve (AUC), where the image area was measured as pixels (i.e., number of pixels within a lipoprotein density interval). This study demonstrated that in a randomized controlled crossover study, both LF and HF ground beef consumption depressed plasma HDL-C and LDL-C concentrations as well as AUC for most HDL and LDL subfractions, providing new insight into the effects of beef consumption on the risk for CVD.

The primary outcome of the current randomized controlled trial was to document the effects of high-fat (HF) ground beef (27 g/patty, 24% fat by weight) and low-fat (LF) ground beef (6 g/patty, 5% fat by weight) on voluntary nutrient intake, and to establish if changes in major macronutrient intake were responsible for any changes we observed in lipoprotein cholesterol concentrations. We hypothesized that consumption of HF ground beef for 5 weeks would reduce the voluntary intake of carbohydrates but have no effect on HDL-C or LDL-C concentrations. Conversely, consumption of LF ground beef for 5 weeks would depress HDL-C and LDL-C concentrations.

## 2. Materials and Methods

### 2.1. Ethics Statements and Participant Recruitment

This randomized, controlled, 2-period crossover trial was conducted in accordance with the Declaration of Helsinki guidelines [8]. The trial was registered at www.clinicaltrials.gov as NCT04841460 accessed on 12 April 2021. All procedures involving human participants were approved by the Texas A&M University Institutional Review Board for use of human participants in research (Protocol number IRB2018-0755). Participants were recruited in October and November 2019, and the ground beef treatments were initiated in February 2020. The final blood samples were collected in July 2020. The study staff were not blinded, but the statistician was blinded to treatment during the initial analyses by identifying the diet conditions as A and B. All subjects were provided with detailed instructions, including potential risks of participation.

**Figure 1:**
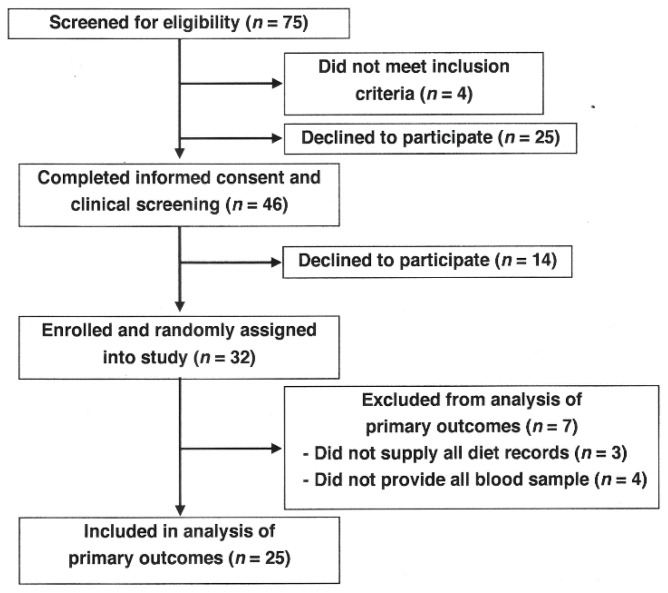
Recruitment flow diagram.

Seventy-five healthy males between the ages of 25 and 60 years participated in one of two informational meetings (Figure 1). Four individuals did not meet the inclusion criteria, and 25 men declined to participate. Forty-six men signed Informed Consent forms, and 14 men later declined to participate. Thirty-two men were assigned at random to two treatment groups, LF and HF ground beef, and were provided test ground beef patties. Seven men who left the study were excluded due to inability to comply (did not provide all diet records, *n* = 3; did not provide all blood samples, *n* = 4), and 25 men completed all phases of the study. 

### 2.2. Inclusion Criteria

**Figure 2:**
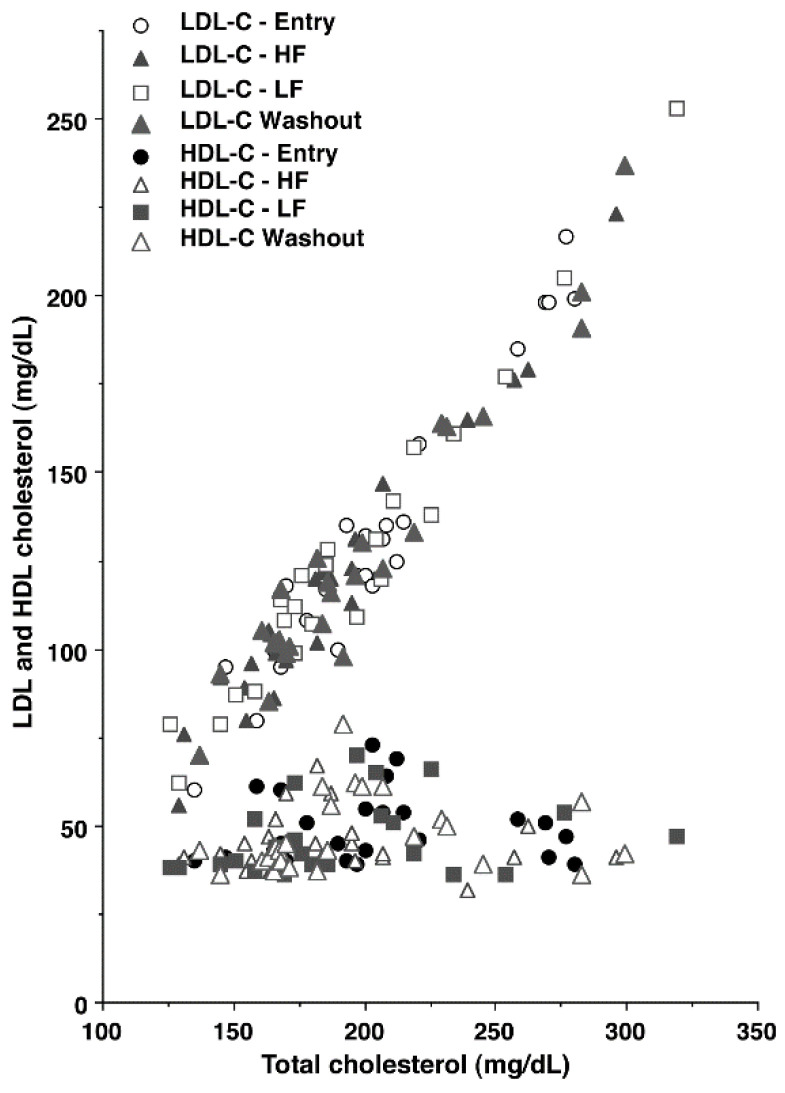
Plasma LDL-C and HDL-C concentrations as a function of plasma total cholesterol concentration. LDL-C, r^2^ = 0.947; HDL-C, r^2^ = 0.022.

The participants had to be non-smoking males, not be on any restrictive diets or cholesterol-lowering medications, and not have a total cholesterol (TC) above 350 mg/dL. The participants were requested not to change their habitual diet or level of physical activity. Due to limited funding, we chose to recruit men only, and not women only or a combination of men and women. Statistical power calculations were based on changes in HDL-C concentration in normocholesterolemic men in response to a ground beef intervention ([2], described below). Unpublished data from previous trials in our laboratory [1,2,3,4] indicated that women had much greater variation in HDL-C concentration at entry (47–120 mg/dL) than men (36–76 mg/dL). The range of HDL-C concentrations at entry in the current study (39–73 mg/dL; Figure 2) was similar to the variation in our previous studies with men. The lesser variation in HDL-C concentration for men improved the power of our statistical analyses, and for this reason, men only were chosen for this trial.

### 2.3. Study Design

The study design was a two-period, randomized crossover design in which each participant completed two 5-week ground beef interventions in a randomly assigned order, with a 4-week washout period [9] between the test periods. All blood samples were taken from the fasting subjects. Four blood samples were drawn at baseline immediately before treatment assignment (entry), immediately after the ground beef interventions, and following the 4-week washout period, immediately before the second ground beef intervention. During the 5-week intervention, the men consumed 5 ground beef patties per week for 5 weeks for each ground beef type, LF and HF (total of 25 patties for each type). The participants were assigned to one of two groups, which were balanced based on plasma HDL-C concentrations measured at the initial screening. Before the first ground beef intervention, the men assigned to the LF group had a mean HDL-C concentration of 51 ± 3 mg/dL, and the men assigned to the HF group had a mean HDL-C concentration of 48 ± 3 mg/dL. Of the 25 men who completed the study, 12 men consumed LF ground beef, and 13 men consumed HF ground beef during the first intervention phase. After the washout period, the groups of 12 and 13 men were rotated to the other test ground beef. 

### 2.4. Source of Ground Beef

The source of raw materials for the production of LF and HF patties were the beef pectoralis muscle and 75:25 coarse ground beef, respectively, purchased from a local supplier (Readfield Meats, Bryan, TX, USA). The muscle raw materials were ground, and 4-ounce (115 g) patties were formed in a patty maker, individually vacuum-packaged, and stored at −20 °C. Prior to the initiation of each phase of the ground beef interventions, each participant received an unlabeled box containing 25 frozen, vacuum-packaged patties. 

Chemical analysis of the ground beef after patty formation indicated that raw LF patties contained 5% fat (6 g fat/patty) and HF patties contained 24% fat (27 g fat/patty) (Table 1). Diet records from previous studies [1,2,3,4] indicated that most study participants pan-broiled the ground beef patties intact, so samples of the LF and HF patties were pan-broiled [10], and total fat and fatty acid composition of the cooked patties were measured. Cooking losses for LF and HF patties were 3% and 41%, respectively. The total lipid and fatty acid composition of the drained pan-broiled patties were used to calculate the daily intake of dietary fats.

### 2.5. Diet Records

The participants were required to complete a 3-day diet record before the diet interventions and once during each intervention to establish nutrient intakes and encourage compliance. Daily intake of major nutrients and dietary exchanges were analyzed by a registered dietitian nutritionist (RDN) using ESHA’s Food Processor Nutrition Analysis software (ESHA, Salem, OR, USA). The participants were trained in the use of myfitnesspal (myfitnesspal.com) to record daily intakes, which were forwarded to the RDN. All participants received instructions from the RDN for the preparation of the ground beef, including recipes, but the participants were not restricted to specific cooking methods for the test ground beef. The RDN also contacted the participants at regular intervals to encourage compliance and provide information about completing diet records.

### 2.6. Body Composition

The body composition of all subjects was assessed at the beginning and at the end of the study using dual-energy X-ray absorptiometry (DXA) (General Electric Lunar Prodigy Advance, Madison, WI, USA). Derived variables of interest from the DXA scans were total body mass, lean body mass, android fat, gynoid fat (all in kg), and percent fat mass. Body mass index (BMI) was calculated for each individual (Table 2).

### 2.7. General Blood Sampling and Analyses

Blood sampling and assay procedures were published previously [11]. On the day of blood sampling (at entry and immediately following the 5-week ground beef interventions and 4-week washout), the subjects were asked to report to the laboratory after an overnight fast (approximately 10 h) restricted to water only. The evening meal was not standardized prior to the study visits; rather, throughout this trial, participants consumed their habitual diets, except for the inclusion of the ground beef patties during the ground beef interventions. Blood was collected after 5 min of seated rest via venipuncture from the antecubital fossa region into serum separator tubes using standard sterile phlebotomy procedures. After collection, the blood was allowed to clot at room temperature for 2 h or chilled at 4 °C for serum and plasma separation, respectively, prior to centrifugation in a refrigerated centrifuge for 20 min (2000× *g*). One serum separator vacutainer was couriered the same day to a commercial Clinical Laboratory Improvement Amendments-certified laboratory for determination of TC, HDL-C, LDL-C, and triglyceride (TG) using standard clinical chemistry analyses. Plasma LDL-C concentration was calculated using the Friedwald equation, which is based primarily on TC (LDL-C = TC − HDL-C − TG/5). Aliquots of serum and plasma from additional vacutainers were transferred into separate 2 mL freezer vials, and the vials were stored frozen at −80 °C until analyzed.

### 2.8. Lipoprotein Density Profiles

Density profiles for circulating lipoproteins were determined by imaging 6 μL serum following NBD-C6-ceramide labeling of lipoproteins, as described [7]. The overall lipoprotein density profile was analyzed as absolute AUC where image area was measured as pixels (i.e., number of pixels within a density interval). Eleven lipoprotein subclasses were identified by their density intervals and quantified by pixel values. The major lipoprotein subclasses were triacylglycerol-rich lipoproteins (TRL; d < 1.019 g/mL), LDL_1_ (d = 1.019–1.023 g/mL), LDL_2_ (d = 1.023–1.034 g/mL), LDL_3_ (d = 1.034–1.044 g/mL), LDL_4_ (d = 1.044–1.055 g/mL), LDL_5_ (d = 1.055–1.063 g/mL), HDL_2b_ (d = 1.063–1.091 g/mL), HDL_2a_ (d = 1.091–1.110 g/mL), HDL_3a_ (d = 1.110–1.133 g/mL), HDL_3b_ (d = 1.133–1.156 g/mL) and HDL_3c_ (d = 1.156–1.179 g/mL) [12]. Lipoprotein density profiles for the participants (not indicated) were essentially identical to those reported previously for men by the co-author Walzem, R.L. [7].

The average percent relative standard deviation in AUC for different lipoprotein subfractions was 4.45% (within-day) and 7.37% (day-to-day). Data were also used to express HDL subfractions as percentages of total HDL AUC:

%LDL_x_ AUC = 100 ∗ %LDL_x_ AUC/total LDL AUC

%HDL_x_ AUC = 100 ∗ %HDL_x_ AUC/total HDL AUC

### 2.9. Statistics

Power calculations were conducted to estimate the required sample size based on HDL-C concentrations from our previous studies with normocholesterolemic men [2]. Plasma HDL-C concentrations increased by 2.8 mg/dL on consumption of 24% fat ground beef [2] compared to habitual diets. Analyses used the following assumptions: power was set at 0.8 and α = 0.05, 2-sided. It was estimated that a sample size of 18 was sufficient to test the hypothesis that HF ground beef would change HDL-C concentrations.

Ground beef effects were analyzed using a repeated measures mixed model to assess the effects of diet (LF vs. HF), sequence (entry, first LF/HF intervention, washout, and second LF/HF intervention), and the diet-by-sequence interaction. Entry BMI and age were included as covariates in the initial model but were insignificant for all dependent variables and were dropped from the final model. The NORM.DIST model of Excel (Microsoft Excel of Mac version 16.16.27) was used to test for normality, and the data were normally distributed. Pairwise comparisons were assessed by Fisher’s Protected LSD method when there was a significant effect of diet or sequence. Associations among plasma lipids were assessed using Pearson’s correlations. Absolute change from baseline was calculated by subtracting measurements taken at entry from post-dietary intervention period values. Data are reported as means ± standard error of the mean (SEM) (*n* = 25 men who completed all phases of the study). Differences among means were considered significant at *p* ≤ 0.05, but tendencies among treatments (*p* ≤ 0.08) will be noted.

## 3. Results

### 3.1. Ground Beef Composition and Participant Nutrient Intake

The LF patties did not lose a detectable amount of fat following pan frying, but the HF patties lost nearly 41% fat after frying. The drained, pan-fried HF patties contained more total SFA, MUFA, and polyunsaturated fatty acids (PUFA) than the pan-fried LF patties (Table 1). Eicosapentaenoic acid (20:5n-3) and docosahexaenoic acid (22:6n-3) were not detectable in the raw or cooked ground beef patties. MUFA:SFA and PUFA:SFA ratios of the LF and HF ground beef were unaffected by pan broiling.

Energy intake did not differ among treatment phases, although energy intake during the LF intervention tended (*p* = 0.071) to be lower than at entry (Table 3). The LF treatment increased % energy from protein relative to entry, washout, and HF treatment (*p* = 0.002). There was a tendency (*p* = 0.062) of the HF treatment to decrease % energy from carbohydrates. The HF treatment increased % energy from fat and SFA relative to entry, washout, and LF treatment (*p* ≤ 0.006).

Protein intake (g/d) tended (*p* = 0.078) to be greatest during the LF treatment (Table 3). Carbohydrate intake was less during the LF intervention than at entry (*p* = 0.048), and dietary fiber intake tended (*p* = 0.062) to be greater at entry and during washout than during the LF and HF treatments. The intakes of soluble fiber, insoluble fiber, total sugars, and added sugars were not affected by the ground beef treatments (*p* ≥ 0.114). Total fat and SFA intakes were greater during the HF treatment than during the washout or LF treatment (*p* ≤ 0.013), but not different from entry. MUFA intake was greatest during the HF treatment (*p* = 0.002) and TFA intake was least during the LF treatment. Intakes of PUFA and linoleic acid were not affected by ground beef consumption (*p* ≥ 0.152), but intake of α-linolenic acid tended (*p* = 0.053) to be depressed during the HF treatment. Cholesterol intake tended (*p* = 0.066) to be lower during washout and during the LF and HF treatments than at entry.

**Figure 3:**
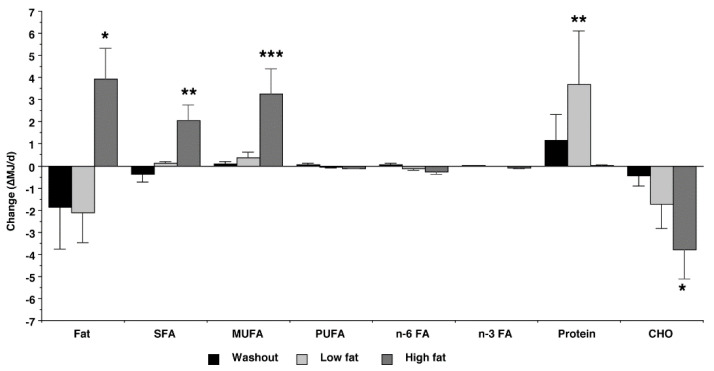
Absolute change (final – entry) in %MJ/d during consumption of ground beef patties initially containing 6 g fat/patty (Low fat) and 27 g fat/patty (High fat). Fat, total fat; SFA, saturated fat; MUFA, monounsaturated fat; PUFA, polyunsaturated fat; CHO, carbohydrate. Bars are means (*n* = 25) with pooled SEM attached. Change statistically different from 0, * *p* ≤ 0.05; ** *p* ≤ 0.01; *** *p* ≤ 0.001.

There were significant (*p* ≤ 0.05) absolute changes from entry for % energy from total fat, SFA, MUFA (which increased), and carbohydrate (which decreased) during the HF intervention (Figure 3). The increase from entry for % energy from protein during the LF intervention also was significant (*p* < 0.05). The mean absolute decreases from entry for cholesterol intake during washout, LF treatment, and HF treatment were 72 mg/d (*p* = 0.119), 95 mg/d (*p* = 0.067), and 101 mg/d (*p* = 0.024), respectively.

### 3.2. Plasma Lipid Concentrations

As indicated above, plasma LDL-C concentration was calculated by the Friedwald equation, which is based primarily on TC (LDL-C = TC − HDL-C − TG/5). For this reason, there was a high correlation between TC and LDL-C concentration (r^2^ = 0.941) (Figure 2). There was greater variation in plasma concentrations of TC (126–319 mg/dL) and LDL-C (56–223 mg/dL) than HDL-C (36–73 mg/dL). At entry, 20 out of 25 participants had TC concentrations < 225 mg/dL. There was no correlation between TC and HDL-C concentration (r^2^ = 0.022) (*p* > 0.25).

**Figure 4:**
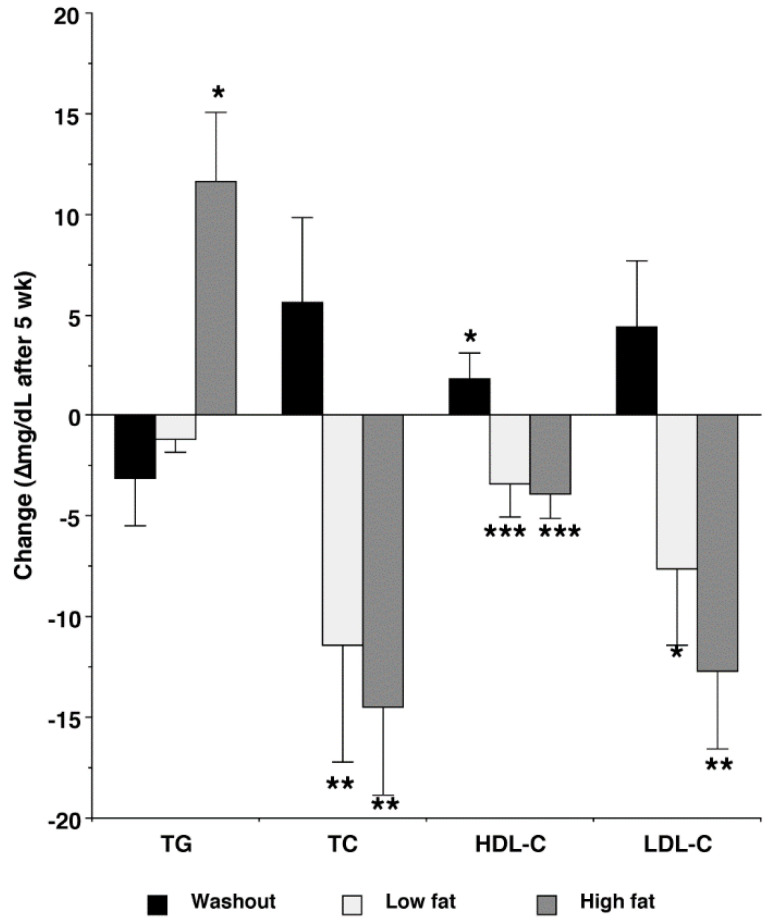
Absolute change (final – entry) in triglyceride and lipoprotein cholesterol following consumption of ground beef patties initially containing 6 g fat/patty (Low fat) and 27 g fat/patty (High fat). TG, triglyceride; TC, total cholesterol; HDL-C, HDL cholesterol; LDL-C, LDL cholesterol. Bars are means (*n* = 25) with pooled SEM attached. Change statistically different from 0, * *p* ≤ 0.05; ** *p* ≤ 0.01; *** *p* ≤ 0.001.

Plasma TG concentration tended (*p* = 0.058) to be greatest following the HF treatment (Table 4). The LF treatment decreased TC, HDL-C, and LDL-C concentrations relative to entry, and the HF treatment decreased TC, HDL-C, and LDL-C concentrations relative to entry and washout. Absolute changes from entry were significant (*p* < 0.05) for TC and HDL-C following the LF treatment, and absolute changes from entry were significant (*p* < 0.05) for TG, TC, HDL-C, and LDL-C following the HF treatment (Figure 4).

### 3.3. Lipoprotein Density Distributions

TRL AUC was greater following the LF intervention than at entry (*p* = 0.050) (Table 4). LDL_4_ AUC represented the greatest proportion of AUC, indicating that LDL was predominantly small and dense. AUC for HDL_3c_ was very low, indicating that HDL_3c_ particles are less numerous than for other HDL subfractions. LDL_1_ and LDL_2_ AUC were not affected by the ground beef treatments, but total AUC and LDL AUC, LDL_3_ AUC, LDL_4_ AUC, and total HDL AUC were lower following washout and the LF and HF treatments than at entry (*p* ≤ 0.024). LDL_5_ AUC was less following the HF intervention than at entry (*p* = 0.041) and HDL_2b_ AUC and HDL_2a_ AUC were less following washout and the LF intervention than at entry (*p* ≤ 0.012). HDL_3a_ AUC was less following the LF and HF interventions than at entry (*p* = 0.002), and HDL_3b_ AUC tended (*p* = 0.058) to be less following the LF treatment than at entry. HDL_3c_ AUC was greater following washout and the LF and HF treatments than at entry (*p* = 0.003). The HF treatment increased %LDL_1_ AUC (*p* = 0.021) and %LDL_2_ AUC (*p* = 0.023), and the LF and HF treatments increased %HDL_3c_ AUC (*p* = 0.001) (Table 5).

### 3.4. Pearson Correlation Coefficients

Plasma HDL-C concentration was negatively correlated with TRL AUC (r = −0.383; *p* < 0.001) and HDL_3b_ AUC (r = −0.277; *p* < 0.01) and was highly, positively correlated with HDL_2b_ AUC (r = 0.914; *p* < 0.0001), HDL_2a_ AUC (r = 0.905; *p* < 0.0001), and total HDL AUC (r = 0.658; *p* < 0.0001) (Table 6). The correlation of HDL-C concentration with HDL_3c_ AUC was not significant (r = −0.046; *p* > 0.25). LDL-C concentration was not correlated with TRL AUC (r = −0.208; *p* > 0.05) but was positively correlated with LDL_1_ AUC (r = 0.322; *p* < 0.001), LDL_2_ AUC (r = 0.600; *p* < 0.0001), LDL_3_ AUC (r = 0.731; *p* < 0.0001), LDL_4_ AUC (r = 0.736; *p* < 0.0001), LDL_5_ AUC (r = 0.251; *p* < 0.05), and total LDL AUC (r = 0.911; *p* < 0.0001).

## 4. Discussion

### 4.1. Energy and Macronutrient Intake and Composition of Patties

The primary outcome of this study was to document which voluntary changes in macronutrient intake were responsible for any observed changes in lipoprotein cholesterol concentrations. The current study confirmed significant increases in % energy from total fat, saturated fatty acids, and monounsaturated fatty acids during the HF treatment. We also calculated absolute change from baseline as previously reported by others [13,14,15,16], and there was an absolute increase from entry for % energy from total fat, saturated fatty acids, and monounsaturated fatty acids during the HF treatment and an increase in % energy from protein during the LF treatment. These findings indicate that the participants voluntarily altered macronutrient intake in response to the fat content of the ground beef treatments.

The calculations of daily fat intake during the LF and HF interventions assumed that, regardless of the method of cooking, drippings were not included in the final food product. We previously reported that pan-broiled, 25% fat ground beef lost 44–49% fat, depending on the degree of doneness [17], and the 41% fat loss of the 24% fat, HF ground beef following pan broiling was similar to our previous results. We also assumed that the fatty acid composition (g fatty acid/100 g total fatty acids) of the pan-fried ground beef patties (drippings not included) would be similar to the composition of raw patties, based on earlier studies from this laboratory [17,18]. As indicated above, the MUFA:SFA and PUFA:SFA ratios were similar in raw and pan-broiled LF and HF patties, indicating that neither monounsaturated fatty acids nor polyunsaturated fatty acids were preferentially lost during cooking. Additionally, polyunsaturated fatty acid intake during ground beef intake was unchanged, indicating that changes observed in lipoprotein cholesterol subfractions were not due to changes in polyunsaturated fatty acid intake.

Many randomized studies have provided set guidelines for nutrient intake during the treatment phases and/or provided baseline diets before initiating dietary interventions [13,14,15,16]. The design of previous studies [1,2,3,4] and the current study differ in that we tested the effects of ground beef interventions on lipoprotein cholesterol concentration following free choice consumption of habitual diets at entry, during the washout period, and during the ground beef interventions. Simple regression over all data (entry, washout, LF, and HF, *n* = 100) indicated that % energy from saturated fatty acids increased as % energy from fat increased (r = 0.810; *p* < 0.0001) and % energy from carbohydrate decreased as % energy from fat increased (r = −0.544; *p* < 0.0001) (data not shown in tabular form). We conclude from these correlations that participants consuming HF ground beef voluntarily reduced carbohydrates in the diet. In addition, increasing beef fat intake markedly increased saturated fatty acid intake, reflecting the fatty acid composition and content in the HF ground beef.

Saturated fatty acid intake by participants in this study exceeded dietary recommendations of less than 10% energy intake from saturated fatty acids; approximately 73% of U.S. males exceed this recommendation [19]. The men in the current study on average consumed 12–13% energy as saturated fatty acids during entry, washout, and LF treatment, and 15% energy as saturated fatty acids during the HF treatment. Maki et al. [20] recently concluded that although the effects of saturated fatty acids on risk factors for CVD remain uncertain, it is prudent to accept the current recommendation [19] of less 10% energy from saturated fatty acids. Despite the reduction in LDL-C concentration caused by the HF intervention in the current study, we also do not recommend exceeding the recommendations [19] for saturated fatty acid intake. We also do not recommend this high level of ground beef intake (i.e., 5 patties/week for 5 weeks). As indicated above, we have chosen to test the effects of ground beef on lipoprotein cholesterol metabolism because total fat and saturated fatty acid composition can be altered accurately during the formulation of ground beef preparations.

### 4.2. High-Density Lipoprotein Cholesterol Concentrations

HDL particles carry out reverse cholesterol transport and possess antioxidative and anti-inflammatory activities through associated proteins and bioactive lipids [21]. These functional properties are variable and can be ascribed to particles of specific diameter ranges [22]. HDL_3_ are small and vary in diameter from approximately 6 to 9.5 nm; HDL_2_ vary in diameter from approximately 9.5 to 13 nm [22,23]. Some have concluded that HDL_3_ includes both the most beneficial and the most detrimental species of HDL [24,25]. Increased amounts of sphingosine-1-P (S1P) and Apo-A1 in HDL_3_ are associated with a robust ability to stabilize LDL against oxidation and attenuate apoptosis in endothelial cells [26]. However, greater plasma concentrations of HDL_3c_-C are associated with greater probability of mortality [27].

Scott et al. [13] reported that a diet containing lean beef (diet = 31% energy as fat; 8% energy as saturated fatty acids) depressed HDL-C concentration in hypercholesterolemic men by 2.4 mg/dL, relative to a high-fat/high saturated fatty acid stabilization diet (diet = 40% energy as fat; 18% energy as saturated fatty acids). Roussell et al. [14] demonstrated that a Beef in an Optimal Lean Diet (BOLD) and a BOLD+ diet (which contained extra beef; both diets = 28% energy as fat; 6% energy as saturated fatty acids) depressed HDL-C concentration in men and women by 3.2 mg/dL, relative to a Healthy American Diet (HAD; diet = 33% energy as fat; 12% energy as saturated fatty acids). In the current study, the LF treatment reduced HDL-C concentration by 3.3 mg/dL, relative to the entry diet, similar to the results of Scott et al. [13] and Roussell et al. [14]. Furthermore, the HF treatment depressed HDL-C concentration by 3.9 mg/dL in spite of the greater total dietary % energy from fat (42%) and saturated fatty acids (15%) relative to the entry diet. It is difficult to understand why the HF treatment elicited results similar to the LF treatment and previous studies [13,14].

### 4.3. Low-Density Lipoprotein Cholesterol Abundance

Each LDL particle contains one apolipoprotein B (apoB), and the larger, less dense LDL_1_ and LDL_2_ are comprised of a greater proportion of cholesterol, whereas the smaller, denser LDL_3_, LDL_4_ and LDL_5_ carry less cholesterol per particle [28]. Reductions in apoB are thought to be as or more beneficial for reducing atherosclerotic CVD risk than reductions in LDL-C concentration [29], and a predominance of small, dense LDL particles (LDL_3-5_) is associated with a greater risk of CVD [29,30,31], in part because apoB comprises a greater proportion of these particles. The reduction in LDL-C concentration reported in the current trial might not be beneficial if caused by a decrease in cholesterol content of the larger, more buoyant LDL particles (LDL_1_ and LDL_2_).

Wang et al. [16] reported that a lower-fat diet decreased TC, LDL-C, and HDL-C concentration relative to an Average American Diet. Moreover, the lower-fat diet caused reductions for cholesterol in the LDL_1_-C and LDL_2_-C subfractions, had no effect on cholesterol content of the LDL_3_-C subfraction, and increased cholesterol content of the LDL_4_-C subfraction [16]. We previously demonstrated that approximately 75% of the total LDL-C is contained in the LDL_1_ plus LDL_2_ subfractions [4]. In addition, ground beef treatments containing 18% or 25% fat increased the cholesterol content of LDL_1_ plus LDL_2_-C subfractions but did not increase the cholesterol content of LDL_3_-C or LDL_4_-C subfractions [4]. We did not measure cholesterol content or apoB amounts in the LDL subfractions in the current study, but we have demonstrated that our ground beef treatments did not affect LDL_1_ or LDL_2_ AUC, but depressed LDL_3-5_ AUC (described below), suggesting that more LDL cholesterol was carried in larger, less dense LDL particles.

### 4.4. Lipoprotein Area under the Curve

NBD-C6-ceramide labels the surface of lipoprotein particles and can be used to indicate particle diameter associated with a specific density interval [32], and can indicate, within that discrete density interval, relative particle abundance in treatment comparisons. Both ground beef interventions depressed HDL_3a_ AUC (hereafter referred to as abundance) and increased HDL_3c_ abundance. The LF treatment also depressed HDL_2b_ and HDL_2a_ abundance. HDL-C concentration was highly correlated with HDL_2b_ abundance (r = 914; *p* < 0.0001) and HDL_2a_ abundance (r = 0.905; *p* < 0.001). A recent study by the co-author Walzem, R.L. reported similar correlations between HDL_2_ abundance and HDL-C concentration [12].

Wang et al. [16] reported that a lower-fat diet depressed the number of small HDL particles in addition to decreasing HDL-C concentration, and we conclude that the LF and HF treatments in the current study also decreased HDL particle abundance. Because HDL_3_ abundance increased from entry, the data suggest that HDL_3a_ and HDL_3b_ particles became denser following HF and LF treatments, shifting their particle densities into the HDL_3c_ density interval. %HDL_3c_ (calculated as a percentage of total HDL AUC) also increased from entry, corroborating a shift from less dense to more dense HDL_3_ particles following the ground beef interventions. As described above, diet analysis during the LF and HF interventions indicated that our participants consumed a much greater % energy from fat/SFA and less % energy from carbohydrates than the BOLD/BOLD+ studies [12,14], which likely explains many of the differences in responses to our dietary interventions.

Small, dense LDL particles have the strongest association with the risk for CVD [33]. Wu et al. [12] reported a reduction of %LDL_4_ (a small, dense LDL subfraction) following the BOLD+ intervention, suggesting that the BOLD+ diet improved the LDL density profile. These results were similar to the current study, in which the LF treatment decreased abundance for small, dense LDL_4_ and the HF treatment decreased abundance for small, dense LDL_4_ and LDL_5_. Wu et al. [12] also reported a decrease in LDL_2_ abundance following the BOLD diet, whereas we observed no change LDL_1_ and LDL_2_ abundance and increased %LDL_1_ and %LDL_2_ abundance following the HF treatment.

### 4.5. Nutrient Intake and Lipoprotein Cholesterol

The LF treatment increased % energy from protein, and the HF treatment increased % energy from fat and saturated fat, and decreased % energy from carbohydrate. However, the LF and HF treatments caused similar reductions in HDL-C and LDL-C concentrations from entry. This seemingly would rule out % energy from protein, fat, or carbohydrate as causative for the reductions in HDL-C and LDL-C concentrations. However, as indicated above, cholesterol intake during washout and the LF and HF treatments was lower than at entry, especially for the HF treatment. Dietary interventions designed to reduce saturated fat intake typically reduced cholesterol intake [13,14,16], and these studies uniformly decreased HDL-C and LDL-C concentrations. We propose that the reduction in cholesterol intake during washout and both ground beef interventions in the current study may have contributed to a general decline in circulating cholesterol concentrations and HDL and LDL subfraction abundance. It is not known why cholesterol intake declined following entry, but we are currently analyzing dietary sources of cholesterol during entry, washout, and ground beef interventions to provide a basis for this observation.

### 4.6. Limitations

Several limitations of the present study should be considered. Because of the nature of the study treatments, the study staff were not blinded, which leads to the potential for bias associated with lack of blinding. The short duration of the intervention, 35 days, may also be considered a limitation, but previous research conducted by the authors has shown that changes in HDL-C and LDL-C concentrations were evident at 5 weeks of making dietary changes [1,2]. The demonstration of a significant carryover effect for some of the lipid AUC values and cholesterol intake should be considered a limitation of not prescribing diets preceding entry or during the washout period. Another limitation is that the participants were men only. Previous trials in our laboratory that included postmenopausal women [3,4] indicated that their responses to ground beef interventions were more variable than for men [1,2]. Moreover, women have greater HDL-C concentration than men, and restricting the study to one gender increased the power of our analyses by reducing the variability inherent between men and women. Finally, we do not recommend the high level of ground beef intake used in this study for the general population.

## 5. Conclusions

In summary, the LF and HF ground beef interventions differently affected voluntary nutrient intake, but the LF and HF treatments similarly depressed lipoprotein cholesterol concentrations. The LF and HF treatments improved the LDL density profile by decreasing abundance for small, dense LDL (LDL_4_ and LDL_5_), and increasing %AUC for large, less dense LDL (LDL_1_ and LDL_2_), relative to entry levels. However, both ground beef interventions increased abundance and %AUC for HDL_3c_, potentially increasing the risk of CVD. Despite frequent moderate ground beef intake (114 g/d), cholesterol intake declined, especially during the HF intervention. For this reason, care should be taken in the interpretation of studies that compare low-fat and low-cholesterol diets to higher-fat and higher-cholesterol diets.

## Figures and Tables

**Table 1:** Fatty acid composition and lipid content of raw and pan-broiled low-fat and high-fat ground beef patties ^1^.

Fatty Acid	Low-Fat		High-Fat	
	Raw	Pan-Broiled	Raw	Pan-Broiled
g fatty acid/114-g beef patty				
Myristic, 14:0	0.16 ± 0.02	0.15 ± 0.01	0.80 ± 0.04	0.46 ± 0.02
Palmitic, 16:0	1.49 ± 0.16	1.45 ± 0.06	6.37 ± 0.35	3.74 ± 0.48
Palmitoleic, 16:1n-7	0.21 ± 0.02	0.20 ± 0.01	0.98 ± 0.05	0.56 ± 0.02
Stearic, 18:0	0.79 ± 0.08	0.77 ± 0.08	3.53 ± 0.19	2.11 ± 0.08
Oleic, 18:1n-9	2.52 ± 0.27	2.17 ± 0.01	9.70 ± 0.53	5.55 ± 0.20
*cis*-Vaccenic, 18:1n-7	0.11 ± 0.01	0.13 ± 0.01	0.52 ± 0.03	0.33 ± 0.01
Linoleic, 18:2n-6	0.27 ± 0.03	0.27 ± 0.03	0.65 ± 0.04	0.41 ± 0.02
α-Linolenic, 18:3n-3	0.01 ± 0.01	0.01 ± 0.01	0.05 ± 0.01	0.02 ± 0.01
Total SFA ^2^	2.44 ± 0.26	2.37 ± 0.23	10.70 ± 0.58	6.32 ± 0.22
Total MUFA ^2^	2.84 ± 0.30	2.50 ± 0.11	11.29 ± 0.71	6.44 ± 0.23
Total PUFA ^2^	0.28 ± 0.03	0.28 ± 0.01	0.71 ± 0.04	0.43 ± 0.02
MUFA:SFA	1.16 ± 0.01	1.06 ± 0.01	1.05 ± 0.01	1.02 ± 0.01
PUFA:SFA	0.11 ± 0.01	0.12 ± 0.01	0.07 ± 0.01	0.07 ± 0.01
Total *trans*-fatty acids ^3^	0.17 ± 0.02	0.16 ± 0.02	1.44 ± 0.08	0.84 ± 0.03
Total lipid per patty ^4^	6.4 ± 0.7	6.2 ± 0.3	26.9 ± 1.5	15.9 ± 0.6

^1^ Values are means ± SE, n = 3 batches of ground beef for each study. ^2^ Total SFA (saturated fatty acids), sum of myristic, palmitic, and stearic acid. Total MUFA (monounsaturated fatty acids), sum of palmitoleic, oleic acid, and cis-vaccenic acid. Total PUFA (polyunsaturated fatty acids), sum of linoleic and α-linolenic acid. Eicosapentaenoic acid (20:5n-3) and docosahexaenoic acid (22:6n-3) were too low to quantify in the ground beef patties. ^3^ Sum of eladic acid (18:1trans-9) and trans-vaccenic acid (18:1trans-11). ^4^ Determined gravimetrically before and after cooking. Includes additional minor fatty acids not included in the table.

**Table 2:** Physiological demographics and DXA-measured body composition for men at baseline and after consumption of ground beef patties (5 patties/week for 5 weeks) initially containing 6 g fat/patty and 27 g fat/patty ^1^.

Item	Entry (Range)	Final	*p*-Values
Age, years	39.9 ± 2.2 (24–58)	40.0 ± 2.2	0.489
Height, cm	177.4 ± 1.4 (168–191)	177.8 ± 1.3	0.409
Weight, kg	97.3 ± 5.0 (76–178)	97.4 ± 5.0	0.499
BMI	31.2 ± 1.8 (23–58)	30.9 ± 1.7	0.461
DXA measurements			
Fat, kg	30.6 ± 3.9 (14–83)	29.6 ± 3.8	0.425
Lean, kg	64.5 ± 1.9 (52–95)	66.9 ± 1.9	0.200
Body fat, %	30.6 ± 3.8 (18–59)	29.6 ± 3.8	0.425
Android fat, %	35.8 ± 2.8 (20–68)	32.0 ± 2.3	0.160
Gynoid fat, %	31.0 ± 2.0 (16–60)	29.7 ± 1.5	0.247

^1^ Values are means ± SEM, n = 25. Range of values for each item is indicated for entry.

**Table 3:** Intakes of major nutrients for men at entry, following washout, and during consumption of ground beef patties initially containing 6 g fat/patty (Low-fat) or 27 g fat/patty (High-fat) ^1^.

Item ^2^	Entry	Washout	Low-Fat	High-Fat	*p*-Value
Total, MJ/d	8.7 ± 0.4	8.1 ± 0.3	7.9 ± 0.4	8.6 ± 0.4	0.071
%MJ/d					
Protein	18.2 ± 0.7 ^b^	19.1 ± 0.9 ^b^	21.9 ± 1.0 ^a^	18.2 ± 1.1 ^b^	0.002
Carbohydrate	42.3 ± 1.7	41.9 ± 1.5	40.9 ± 1.6	38.5 ± 1.7	0.060
Fat	38.4 ± 1.1 ^b^	36.7 ± 1.4 ^b^	36.5 ± 1.4 ^b^	42.4 ± 1.7 ^a^	0.006
SFA ^3^	12.6 ± 0.6 ^b^	12.3 ± 0.5 ^b^	12.6 ± 0.7 ^b^	14.7 ± 0.7 ^a^	0.004
MUFA	6.5 ± 0.6 ^b^	6.7 ± 0.9 ^b^	7.1 ± 0.5 ^b^	9.8 ± 0.9 ^a^	0.003
PUFA	3.6 ± 0.3	3.5 ± 0.4	3.5 ± 0.4	3.4 ± 0.3	0.376
Intake, g/d					
Protein	93.8 ± 5.5	91.3 ± 4.2	102.2 ± 6.2	93.2 ± 7.5	0.078
Carbohydrate	217.4 ± 12.2 ^a^	203.4 ± 9.9 ^ab^	190.5 ± 9.8 ^b^	197.0 ± 12.6 ^ab^	0.048
Dietary fiber	17.8 ± 1.2	17.4 ± 1.5	15.4 ± 0.9	15.3 ± 1.2	0.062
Soluble fiber	0.8 ± 0.2	0.9 ± 0.1	0.9 ± 0.1	0.8 ± 0.1	0.334
Insoluble fiber	2.0 ± 0.4	2.6 ± 0.6	2.5 ± 0.4	2.4 ± 0.4	0.175
Total sugars	68.6 ± 7.0	58.9 ± 5.7	65.6 ± 5.5	64.5 ± 7.9	0.140
Added sugars	8.6 ± 3.1	10.5 ± 3.8	14.6 ± 3.9	14.0 ± 4.3	0.114
Total fat	89.2 ± 5.1 ^ab^	80.2 ± 4.9 ^bc^	76.7 ± 4.4 ^c^	96.6 ± 6.0 ^a^	0.006
SFA	29.2 ± 1.8 ^ab^	26.9 ± 1.8 ^b^	26.5 ± 1.7 ^b^	33.7 ± 2.5 ^a^	0.013
MUFA	15.4 ± 1.6 ^b^	14.1 ± 1.7 ^b^	15.2 ± 1.4 ^b^	22.2 ± 2.2 ^a^	0.002
PUFA	8.1 ± 0.8	7.6 ± 0.9	7.4 ± 1.0	7.6 ± 0.7	0.293
*trans*-Vaccenic acid	0.7 ± 0.2 ^a^	0.5 ± 0.1 ^ab^	0.3 ± 0.1 ^b^	0.6 ± 0.2 ^ab^	0.011
Linoleic acid	5.9 ± 0.8	5.4 ± 0.7	5.1 ± 0.9	4.9 ± 0.5	0.152
α-Linolenic acid	0.7 ± 0.1	0.7 ± 0.1	0.6 ± 0.1	0.5 ± 0.1	0.053
Cholesterol, mg/d	415 ± 57	335 ± 34	328 ± 39	314 ± 33	0.066

^1^ Values are means ± SEM, n = 25. ^2^ Data were derived from 3-day diet records collected during each test period, to include one weekend day. ^3^ SFA = saturated fatty acids, primarily palmitic and stearic acid. MUFA = monounsaturated fatty acids, primarily oleic acid. PUFA = polyunsaturated fatty acids, primarily linoleic acid and α-linolenic acid. A repeated measures mixed model was used to assess the effects of diet, sequence, and the diet-by-sequence interaction. ^ab^ Pairwise comparisons were assessed by Fisher’s Protected Least Squares Difference method when there was a significant diet effect.

**Table 4:** Lipoprotein cholesterol and triglyceride concentrations and area under the curve for lipoprotein subfractions for men at entry and after consumption of ground beef patties initially containing 6 g fat/patty (Low-fat) and 27 g fat/patty (High-fat) ^1^.

Item	Entry	Washout	Low-Fat	High-Fat	*p*-Value
TG, mg/dL ^2^	111.4 ± 9.6	114.6 ± 10.9	110.1 ± 8.6	123.0 ± 11.1	0.058
TC, mg/dL	203.0 ± 8.2 ^a^	197.4 ± 8.5 ^ab^	191.5 ± 8.8 ^bc^	188.5 ± 8.1 ^c^	0.008
HDL cholesterol, mg/dL	49.3 ± 2.0 ^a^	47.6 ± 2.2 ^ab^	46.0 ± 1.9 ^bc^	45.4 ± 1.8 ^c^	0.001
LDL cholesterol, mg/dL	131.2 ± 8.1 ^a^	126.9 ± 7.9 ^ab^	123.6 ± 8.5 ^bc^	118.5 ± 7.5 ^c^	0.011
Total AUC	1920 ± 46 ^a^	1844 ± 44 ^b^	1815 ± 51 ^b^	1811 ± 48 ^b^	0.003
TRL AUC	150 ± 14 ^b^	165 ± 18 ^ab^	173 ± 17 ^a^	159 ± 15 ^ab^	0.050
Total LDL AUC	827 ± 34 ^a^	772 ± 31 ^b^	752 ± 34 ^b^	746 ± 32 ^b^	0.001
LDL_1_ AUC	29 ± 1	28 ± 2	29 ± 2	30 ± 2	0.225
LDL_2_ AUC	50 ± 3	48 ± 28	48 ± 3	48 ± 3	0.450
LDL_3_ AUC	170 ± 11 ^a^	159 ± 9 ^b^	155 ± 12 ^b^	152 ± 9 ^b^	0.024
LDL_4_ AUC	387 ± 25 ^a^	354 ± 22 ^b^	341 ± 24 ^b^	341 ± 22 ^b^	0.002
LDL_5_ AUC	189 ± 15 ^a^	181 ± 15 ^ab^	178 ± 10 ^ab^	173 ± 13 ^b^	0.041
Total HDL AUC	942 ± 29 ^a^	906 ± 25 ^b^	884 ± 301 ^b^	905 ± 28 ^b^	0.003
HDL_2b_ AUC	225 ± 18 ^a^	209 ± 174 ^b^	211 ± 20 ^b^	212 ± 18 ^ab^	0.012
HDL_2a_ AUC	245 ± 11 ^a^	230 ± 8 ^b^	225 ± 11 ^b^	234 ± 10 ^ab^	0.007
HDL_3a_ AUC	278 ± 8 ^a^	268 ± 5 ^ab^	259 ± 7 ^b^	264 ± 6 ^b^	0.002
HDL_3b_ AUC	135 ± 4	133 ± 4	128 ± 4	130 ± 5	0.058
HDL_3c_ AUC	58 ± 1 ^b^	64 ± 1 ^a^	62 ± 1 ^a^	64 ± 2 ^a^	0.003

^1^ Values are means ± SEM, n = 25 in a crossover design. ^2^ AUC, area under the curve (unitless) of all lipid-rich fractions; TRL, triglyceride-rich lipids; LDL, low-density lipoprotein; HDL, high-density lipoprotein. A repeated measures mixed model was used to assess the effects of diet and sequence, and the diet-by-sequence interaction. ^abc^ Pairwise comparisons were assessed by Fisher’s Protected Least Squares Difference method when there was a significant diet effect.

**Table 5:** Percent area under the curve for lipoprotein fractions for men at entry and after consumption of ground beef patties initially containing 6 g fat/patty (Low-fat) and 27 g fat/patty (High-fat) ^1^.

Item	Entry	Washout	Low-Fat	High-Fat	*p*-Value
%LDL_1_ AUC ^2^	3.7 ± 0.2 ^b^	3.7 ± 0.2 ^b^	4.0 ± 0.2 ^ab^	4.1 ± 0.2 ^a^	0.021
%LDL_2_ AUC	6.1 ± 0.3 ^b^	6.3 ± 0.3 ^ab^	6.4 ± 0.3 ^ab^	6.5 ± 0.2 ^a^	0.023
%LDL_3_ AUC	20.6 ± 0.9	20.5 ± 0.8	20.5 ± 0.9	20.2 ± 0.7	0.534
%LDL_4_ AUC	46.3 ± 1.8	45.7 ± 1.7	45.0 ± 1.9	45.4 ± 1.6	0.229
%LDL_5_ AUC	23.3 ± 1.8	23.7 ± 1.8	24.1 ± 1.7	23.7 ± 1.8	0.325
%HDL_2b_ AUC	23.3 ± 1.2	22.5 ± 1.2	23.0 ± 1.3	22.8 ± 1.2	0.164
%HDL_2a_ AUC	25.9 ± 0.5	25.4 ± 0.4	25.3 ± 0.5	25.7 ± 0.5	0.125
%HDL_3a_ AUC	29.8 ± 0.7	29.9 ± 0.7	29.8 ± 0.9	29.6 ± 0.7	0.256
%HDL_3b_ AUC	14.7 ± 0.6	15.0 ± 0.5	14.8 ± 0.6	14.7 ± 0.6	0.472
%HDL_3c_ AUC	6.3 ± 0.2 ^b^	7.2 ± 0.3 ^a^	7.1 ± 0.3 ^a^	7.3 ± 0.3 ^a^	0.001

^1^ Values are means ± SEM, *n* = 25 in a crossover design. ^2^ AUC, area under the curve of lipid-rich subfractions; LDL, low-density lipoprotein; HDL, high-density lipoprotein. %LDL_x_ AUC = 100 ∗ (%LDL_x_ AUC/total LDL AUC); %HDL_x_ AUC = 100 ∗ (%HDL_x_ AUC/total HDL AUC). A repeated measures mixed model was used to assess the effects of diet and sequence, and the diet-by-sequence interaction. ^ab^ Pairwise comparisons were assessed by Fisher’s Protected Least Squares Difference method when there was a significant diet effect.

**Table 6:** Pearson correlations among plasma HDL-C and LDL concentrations and area under the curve for HDL and LDL subfractions ^1,2^.

Item	Area under the Curve						
	**TRL**	**HDL_2b_**	**HDL_2a_**	**HDL_3a_**	**HDL_3b_**	**HDL_3c_**	**Total HDL**
HDL-C	−0.383 ***	0.914 ****	0.905 ****	0.128	−0.277 **	−0.046	0.658 ****
	**TRL**	**LDL_1_**	**LDL_2_**	**LDL_3_**	**LDL_4_**	**LDL_5_**	**Total LDL**
LDL-C	−0.208	0.332 ***	0.600 ****	0.731 ****	0.736 ****	0.251 *	0.911 ****

^1^ Values are simple correlation coefficients, *n* = 100 (entry, washout, low-fat and high-fat values). ^2^ HDL-C, high-density lipoprotein cholesterol; LDL, low-density lipoprotein cholesterol; Total HDL and LDL, total area under the curve for HDL and LDL subfractions, respectively. * *p* < 0.05; ** *p* < 0.01; *** *p* < 0.001; **** *p* < 0.0001.

## Data Availability

The data presented in this study are available on request from the corresponding author. The data are not publicly available as no appropriate public database is available.

## Abbreviations

AUC, area under the curve; BMI, body mass index; CVD, cardiovascular disease; DXA, dual-energy X-ray adsorptiometry; HDL-C, high-density lipoprotein cholesterol; HF, high-fat; LDL-C, low-density lipoprotein cholesterol; LF, low-fat; MUFA, monounsaturated fatty acids; PUFA, polyunsaturated fatty acids; RDN, registered dietitian nutritionist; SFA, saturated fatty acids; TG, triglycerides; TFA, *trans*-fatty acids.
